# A Bioinformatics-Assisted Review on Iron Metabolism and Immune System to Identify Potential Biomarkers of Exercise Stress-Induced Immunosuppression

**DOI:** 10.3390/biomedicines10030724

**Published:** 2022-03-21

**Authors:** Diego A. Bonilla, Yurany Moreno, Jorge L. Petro, Diego A. Forero, Salvador Vargas-Molina, Adrián Odriozola-Martínez, Carlos A. Orozco, Jeffrey R. Stout, Eric S. Rawson, Richard B. Kreider

**Affiliations:** 1 Research Division, Dynamical Business & Science Society—DBSS International SAS, Bogota 110311, Colombia; lymoreno@mdanderson.org (Y.M.); jlpetro@dbss.pro (J.L.P.); 2 Research Group in Biochemistry and Molecular Biology, Faculty of Science and Education, Universidad Distrital Francisco José de Caldas, Bogota 110311, Colombia; 3 Research Group in Physical Activity, Sports and Health Sciences (GICAFS), Universidad de Córdoba, Montería 230002, Colombia; 4 Sport Genomics Research Group, Department of Genetics, Physical Anthropology and Animal Physiology, Faculty of Science and Technology, University of the Basque Country (UPV/EHU), 48940 Leioa, Spain; adrian.odriozola@ehu.eus; 5 Health and Sport Sciences Research Group, School of Health and Sport Sciences, Fundación Universitaria del Área Andina, Bogotá 111221, Colombia; dforero41@areandina.edu.co (D.A.F.); corozco35@areandina.edu.co (C.A.O.); 6 Faculty of Sport Sciences, EADE-University of Wales Trinity Saint David, 29018 Málaga, Spain; salvadorvargas@eade.es; 7 kDNA Genomics^®^, Joxe Mari Korta Research Center, University of the Basque Country UPV/EHU, 20018 Donostia, Spain; 8 Physiology of Work and Exercise Response (POWER) Laboratory, Institute of Exercise Physiology and Rehabilitation Science, University of Central Florida, Orlando, FL 32816, USA; jeffrey.stout@ucf.edu; 9 Department of Health, Nutrition and Exercise Science, Messiah University, Mechanicsburg, PA 17055, USA; erawson@messiah.edu; 10 Exercise & Sport Nutrition Laboratory, Human Clinical Research Facility, Department of Health & Kinesiology, Texas A&M University, College Station, TX 77843, USA; rbkreider@tamu.edu

**Keywords:** ferritins, hemeproteins, transferrin receptor, metabolic networks and pathways, immune system, physiological stress response, exercise, allostasis

## Abstract

The immune function is closely related to iron (Fe) homeostasis and allostasis. The aim of this bioinformatics-assisted review was twofold; (i) to update the current knowledge of Fe metabolism and its relationship to the immune system, and (ii) to perform a prediction analysis of regulatory network hubs that might serve as potential biomarkers during stress-induced immunosuppression. Several literature and bioinformatics databases/repositories were utilized to review Fe metabolism and complement the molecular description of prioritized proteins. The Search Tool for the Retrieval of Interacting Genes (STRING) was used to build a protein-protein interactions network for subsequent network topology analysis. Importantly, Fe is a sensitive double-edged sword where two extremes of its nutritional status may have harmful effects on innate and adaptive immunity. We identified clearly connected important hubs that belong to two clusters: (i) presentation of peptide antigens to the immune system with the involvement of redox reactions of Fe, heme, and Fe trafficking/transport; and (ii) ubiquitination, endocytosis, and degradation processes of proteins related to Fe metabolism in immune cells (e.g., macrophages). The identified potential biomarkers were in agreement with the current experimental evidence, are included in several immunological/biomarkers databases, and/or are emerging genetic markers for different stressful conditions. Although further validation is warranted, this hybrid method (human-machine collaboration) to extract meaningful biological applications using available data in literature and bioinformatics tools should be highlighted.

## 1. Introduction

Iron (Fe) is one of the most abundant metals on earth and is an essential trace element for most of the different living forms. In human physiology, Fe is the most abundant microelement in the organism [1]. With a relative atomic mass of 55.847 and atomic number 26, natural Fe is a stable mixture of nuclides with corresponding relative masses of 54 (5.8%), 56 (91.7%), 57 (2.2%), and 58 (0.3%) [2]. This metal facilitates electron transfer reactions in the respiratory chain and is important in mitochondrial energy metabolism. Furthermore, Fe is an important component of hemoglobin (needed to carry oxygen and other chemical species) and myoglobin (stores oxygen in the muscle and releases it when needed during contraction), besides several other enzymes [3]. Fe is indispensable for the formation and function of erythrocytes due to their high hemoglobin content [4].

The average amount of Fe in our body is about 4.5 g, representing 0.01% of body mass. Reserves of this mineral are found in the liver, spleen, and bone marrow, mainly in the form of ferritin—a complex formed by ferritin heavy chain (FTH1) and ferritin light chain (FTL)—and as hemosiderin to a lesser extent [5]. It is worth noting that there are two types of Fe from the diet: heme and non-heme Fe. While heme-Fe comes exclusively from animal food, given that it participates in the structure of the heme group (forming a coordination complex attached to porphyrin), non-heme Fe is present in both plants and animal food. It should be noted that heme-Fe is absorbed more efficiently than the non-heme [6]. The recommended dietary allowance (RDA) for Fe in all age groups of men and postmenopausal women is 8 mg per day; however, this value increases to 18 mg per day in premenopausal women due to menstrual losses [7]. Furthermore, the RDA for Fe rises to 27 mg per day during pregnancy and decreases in the lactation period (10 mg in females aged 14–18 years and 9 mg in women aged 19–50 years) [8]. It is noteworthy to mention that the RDA for vegetarians and/or vegans is about 1.8 times higher than the omnivorous population [7]. The median dietary intake of Fe is approximately 16–18 mg·day^−1^ for men and 12 mg·day^−1^ for women, while the tolerable upper intake level for adults is 45 mg·day^−1^, considering gastrointestinal distress as an adverse effect [9]. The bioavailability of Fe is 14–18% in populations that consume a mixed diet and 5–12% in people with vegetarian diets [10]. Fe bioavailability in a healthy adult is between 10–15% from the diet, highlighting the absorption at the intestinal mucosa level as the main point of regulation [11,12]. Intriguingly, the human body has no controlled mechanisms for the excretion of Fe, and the levels are balanced by regulating Fe absorption [13] at the cellular and the systemic level [14]; hence, a daily quantity of 1–2 mg of intestinal Fe absorption is required for maintaining normal Fe concentrations [12]. Notwithstanding this, based on isotopic and chemical analysis, proposed mechanisms for Fe excretion encompass sloughed mucosal cells, intestinal epithelium turnover, skin exfoliation, and other blood losses (e.g., menstruation) [15,16,17]. In addition, it is proposed that Fe excretion occurs at a basal rate regardless of Fe deficiency or excess [2,17].

**Figure 1:**
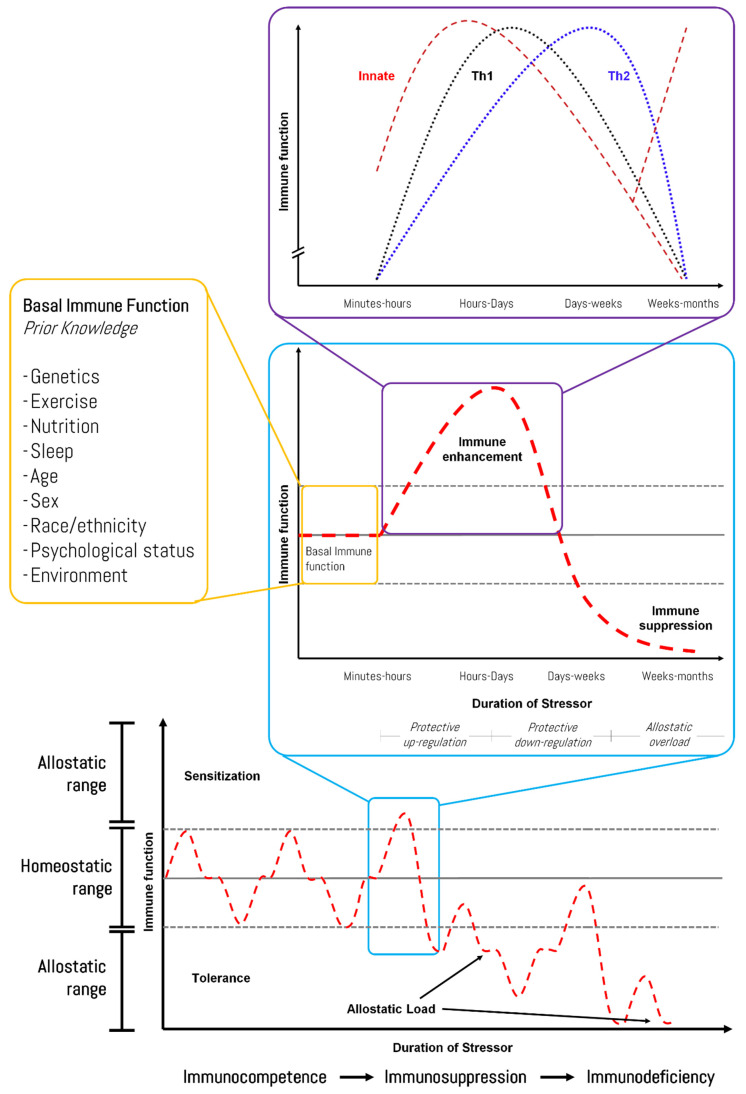
Representation of the changes in the immune function in response to stress. The figure shows the response pattern of immune activity to distinguish between allostatic load in the normal life cycle and allostatic overload that exceeds the capacity of the biological system to cope. See the previous paragraphs of the manuscript for further rationale. Source: designed by the authors (D.A.B.) based on published materials [29,50,51,52].

Humans, among other mammals, need to fulfill their energy and micronutrients requirements for adequate functioning in cases of physiological stress [18]. Based on Selye [19], stress can be defined as the response to any external and/or internal challenge (i.e., stressors) which produces extreme disturbances (mediated by receptors and secondary messengers) beyond the normal physiological function (arousal) in a given biological system. This over-activation triggers signaling pathways that aim to control the stress and reach homeostasis through negative feedback and feedforward motifs at the cellular and systemic levels [20,21,22]. Extreme and constant over-activation modifies several, if not all, parameters of the biological system to cope appropriately with chronic demands and maintain stability—even outside of the normal homeostatic range [23]. Thus, the biological system resets the primary mediators of the physiological response at a new set point that is different from the normal (homeostatic) operating level in a process that is called allostasis or “stability through change” [24]. The cost the biological systems have to pay for being forced to adapt to this new set point has been defined as allostatic load [25]. In the context of physiological regulation and adaptation, the allostasis model represents the current health paradigm to anticipate stress-mediated needs (e.g., timely provision of food, adequate environmental conditions) and understand the process of diseases as constantly changing biological situations [26,27]. Multiple mechanisms are involved in the appropriate response to stress and the development of allostatic status, with the immune system—innate and adaptive immunity—as an important regulator (immunocompetence). Immune activity should be enhanced in response to short-term transient stress (lasting minutes to hours) to ensure survival and optimal function of the biological system; however, immunity tends to be diminished if long-term stress continues over days to months [28]. This down-regulation of the immune system (immunosuppression) cannot be rapid since the biological system is a diffuse network of cells and tissues that require the reset of regulatory parameters to redirect resources towards activities that are more immediately valuable to survival (allostatic load) [29]. The allostatic load can increase dramatically if the system has superimposed on it additional loads that exceed the capacity to cope (e.g., inherited immunodeficiency disorders, HIV infection, cancer, malnutrition, drug-induced side effects [including steroids, ciclosporin, and rapamycin] [30]), in so-called allostatic overload (immunodeficiency) [31]. Figure 1 shows the response pattern of the immune function to different duration/intensity stressors, although individual variation (i.e., prior knowledge) should be considered.

Physical exertion is a common stressor that has been evaluated in many models [32]. As expected, it might benefit or threaten a biological system based on the intensity of the stimulus (exercise dosage) [33,34]. Interestingly, adequate doses of physical exercise and increased physical activity levels have been associated with lower allostatic load [27,35]. Mechanisms that provide an adequate response to physical stress factors, such as strenuous or vigorous exercise, involve molecular regulators, such as heat shock proteins [36] and immune function activation. The availability of Fe plays a key role and is regulated by several pathways and proteins [37]. Data accumulated from several studies have shown that exercise itself would not lead to a true Fe deficiency [38,39,40], or so-called “sports anemia,” in a healthy athlete with adequate daily Fe intake. Hence, the greatest predisposition to Fe-deficiency anemia in young female athletes may not be exercise itself, but probably low energy availability, inadequate dietary choices, reduced Fe intake, and menstruation [41,42]. These factors may also induce Fe deficiency or anemia in the general population [43]. However, recognition of such scenarios has also been seen in male athletes, contributing to the more inclusive concept of relative energy deficiency in sport (RED-S) syndrome [44] as an expanded concept of the female athlete triad. In this sense, it is important to monitor the responses of the immune system to physical exercise given that it is highly linked to Fe metabolism [45]. Although the exact mechanism is still unknown, there are certain molecular biomarkers associated with adaptive regulation processes and Fe regulation (e.g., increase in hepcidin [HAMP] levels) [46,47]. Identifying novel biomarkers during these stress-related immune responses might help with therapeutic guidance and in monitoring the allostatic load [48,49], for example, during the athletes’ preparation for physical competition. Guidi et al. [27] have recently suggested that an integrated approach that includes biological markers and clinical monitoring to assess allostatic load is highly important to track responses to stress. Therefore, the aim of this bioinformatics-assisted review is twofold; (i) to update the current knowledge of Fe metabolism and its relationship to the immune system with a special emphasis on the potential mechanisms of action and signaling pathways, and (ii) to perform a prediction analysis of regulatory network hubs that might serve as potential biomarkers during stress-induced immunosuppression with exercise as a stress model.

## 2. Methods

### 2.1. Search Strategy and Information Sources

The searching process of the scientific literature was carried out using the free terms “iron,” “metabolism,” and “immune system” through the databases PubMed/MEDLINE and Science Direct. Further papers were sought by hand-searching in Google Scholar. 

### 2.2. Manual Curation and Bioinformatics-Assisted Review 

The literature review followed the basic framework for integrative reviews described by Whittemore and Knafl [53], which allows for the inclusion of quantitative and qualitative studies. In addition, we used the optimized methodology established by Hopia et al. [54] for the evaluation and analysis of scientific publications, including problem formulation, literature search, evaluation, analysis, and presentation of findings. 

Bioinformatics-assisted review is a new approach that has been recently developed by Bonilla et al. [55] to address the lack of systematization in narrative reviews that aim to update and/or analyze potential mechanisms of action. It also allows extracting experimentally validated and biologically important information for a given biological phenomenon under a systems biology approach which would otherwise be cumbersome to extract manually. Considering the importance of the various data sources, a high-level of manual curation and reproducibility (open source) were required. Several bioinformatics databases/repositories were used for cross-referencing, functional annotation, and to enrich biological significance, including UniProtKB (https://www.uniprot.org/, accessed on 17 June 2021), PDB (https://www.rcsb.org/, accessed on 17 June 2021), Ensembl (https://www.ensembl.org/index.html, accessed on 17 June 2021), The Gene Ontology (GO) Resource (http://geneontology.org/, accessed on 17 June 2021) and the BioGPS—Gene Portal System (http://biogps.org/, accessed on 17 June 2021). The data search/enrichment was performed between April and June 2021, although an updated search was conducted prior to manuscript submission. Gene/protein prioritization was based on pathways and regulation of Fe metabolism (synthesis and transport). Manual curation of literature and bioinformatics data was performed by one author (D.A.B.), with experience in the extraction of kinase-substrate interactions from the literature [55,56], who also participated in the data extraction for the development of the Kinase Enrichment Analysis version 2 by the Ma’ayan Laboratory (capstone project) [57]. A second author, with extensive experience in bioinformatics and systems biology (D.A.F.), revised and supervised the analytics workflow.

### 2.3. Identification of Potential Biomarkers

**Figure 2:**
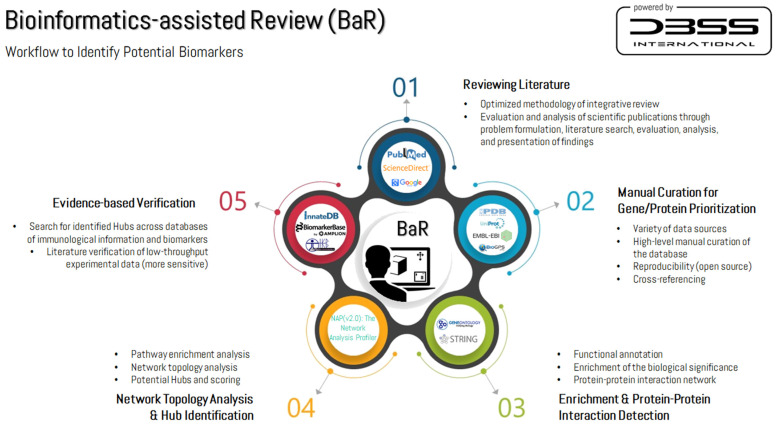
Overview of the bioinformatics-assisted review workflow to identify potential biomarkers.

The prioritized gene/proteins identified in the manual curation were submitted to the Search Tool for the Retrieval of Interacting Genes (STRING, https://string-db.org/, accessed on 17 June 2021) [58] to build a protein/protein interactions network (PPIN). All STRING scores rank from 0 to 1, with 1 being the highest possible confidence. A score of 0.5 would indicate that roughly every second interaction might be erroneous (i.e., a false positive). The following options were activated in the STRING tool to obtain the PPIN: (i) search—by multiple proteins; (ii) network type—full STRING network; (iii) meaning of network edges—evidence; (iv) minimum required interaction score—high confidence (0.700); and (v) max number of interactors to show—1st shell = no more than five interactors, and 2nd shell = no more than five interactors. To cluster the most similar nodes of the network into an easily distinguishable function-based classification (e.g., immune system regulation), we used the Markov cluster algorithm for graphs, which is based on simulation of stochastic flow in the obtained graph. The inflation factor was set at 1.5 to balance sensitivity and selectivity. STRING and GO have been complementarily used in previous studies as the main sources of data for constructing network models and providing biological outputs for the PPIN, respectively [59,60]. The identification of hub nodes was based on network topology and STRING average score. Network topology analysis was performed using the Network Analysis Profiler v2.0 (http://bib.fleming.gr:3838/NAP/, accessed on 2 July 2021) [61]. Data representation in network models was utilized as the prioritization approach [62], although we also implemented manual curation of the STRING data (interactions in tabular form) utilizing literature verification to improve reliability [63]. The results were verified by contrasting the network hubs to the individual experimental reports available in the literature using exercise-induced immunosuppression as a model example. We also searched in online databases for (i) experimentally-verified biological entities involved in the immune response of humans, such as InnateDB (available at http://innatedb.sahmri.com/index.jsp, accessed on 7 November 2021) [64], and the Immunome Knowledge Base (available at http://structure.bmc.lu.se/idbase/ikb/, accessed on 7 November 2021) [65]; and (ii) biomarkers at BiomarkerBase (available at https://www.biomarkerbase.com/, accessed on 7 November 2021) and MarkerDB (available at https://markerdb.ca/, accessed on 7 November 2021) [66]. These tools were accessed between September and October 2021. Figure 2 shows the general workflow of this study. 

## 3. Iron Uptake and Metabolism

Fe in the body can be found as non-heme Fe and heme-Fe. Non-heme Fe comes from both plant and animal-derived foods and is absorbed in a small proportion (3–8%). The presence of vitamin C increases its absorption and other organic acids that transform non-heme Fe from its ferric (Fe^3+^) to ferrous (Fe^2+^) state. The last is directly absorbed at the level of the intestinal mucosa or other cells [67]. Since non-heme Fe reaches the intestine primarily in the Fe^3+^ state, it needs to be reduced to Fe^2+^ by the action of ferrireductases. In the duodenum, this reduction is carried out mainly by cytochrome b reductase 1 (CYBRD1) [68]. It is hypothesized that there are other ferrireductases in intestinal enterocytes, since it has been shown that mice lacking CYBRD1 do not suffer from impaired Fe absorption [69]. Fe^2+^ finally enters duodenal epithelial cells through the natural resistance-associated macrophage protein 2 (NRAMP, also known as divalent metal ion transporter 1 or DMT1).

On the other hand, heme-Fe participates in the structure of the heme group, where Fe is part of a coordination complex attached to porphyrin and comes exclusively from animal food as an easily absorbed source [70]. This is part of hemoglobin, myoglobin, and other enzymes, such as cytochromes, catalases, and peroxidases, that participate in oxidative processes [71]. Whereas the interaction with haptoglobin and CD163 mediates the lysoendosomal trafficking of hemoglobin from plasma to cells [72], heme-Fe can be transported into duodenal cells by solute carrier family 46 member 1 (SLC46A1, also known as the proton-coupled folate transporter) [73]. Once it reaches the enterocyte endosomal membrane, the heme group is degraded by the action of heme oxygenases (HMOX1 and HMOX2), and Fe^2+^ is released to the cytosol. Alternatively, the scavenger receptor class A member 5 (SCARA5) mediates cellular uptake of ferritin-bound Fe by stimulating ferritin endocytosis from the cell surface with consequent Fe delivery within the cell [74]. The functional molecule of ferritin forms a roughly spherical shell of 24-mer FTL/FTH1 with a diameter of 12 nm and contains a central cavity into which the insoluble mineral Fe core is deposited (≈4000–4500 Fe atoms) [75]. Damaged ferritin is called hemosiderin, which is functionally defined as insoluble cellular Fe [76]. Depending on human body requirements, cytosolic Fe^2+^: (i) can be stored in the enterocyte by binding to FTL/FTH1, the intracellular Fe storage protein complex [75]; (ii) distributed around the cell, mediated by poly(rC)-binding proteins (PCBPs, also known as intracellular Fe chaperones [77,78]); or, (iii) released into the bloodstream via solute carrier family 40 member 1 (SLC40A1, also known as ferroportin) [79]. Thus, once within the cell, Fe^2+^ can be stored as ferritin, bind to chaperones (e.g., PCBPs) for travelling to other organelles (e.g., mitochondria [80]), or even be regulated at the transcriptional (less-known) and post-transcriptional level (i.e., the IREB/IRE system) to control its uptake, storage and export. The iron-responsive element-binding proteins (IREBs, also known as Fe-sensing proteins or iron-regulatory proteins, IRPs) and iron-responsive elements (IREs, which are 30-nucleotide long RNA motifs that form special stem-loop structures) create the so-called IREB/IRE system, which enables the cell to minimize or maximize its Fe transport or storage according to need [81]. The binding of the IREBs to the IREs can be at either the 3′-untranslated region (UTR) or 5′-UTR of a respective mRNA to control its translation [82]. Interestingly, binding to the 5′-UTR blocks translation while binding to the 3′ UTR stabilizes the mRNA against endonuclease cleavage [83]. This key hub of intracellular Fe metabolism post-transcriptionally regulates many genes (e.g., *FTL/FHL1*, *SLC40A1*, *SLC11A2*) [84] by specifically binding to the conserved IREs located in the UTRs of mRNAs [85]. Description of the genes/transcripts that may be affected by the IREB/IRE system and how this binding impacts the translation of these transcripts can be found in the publications by Zhang et al. [84] and Khan et al. [81], respectively.

**Figure 3:**
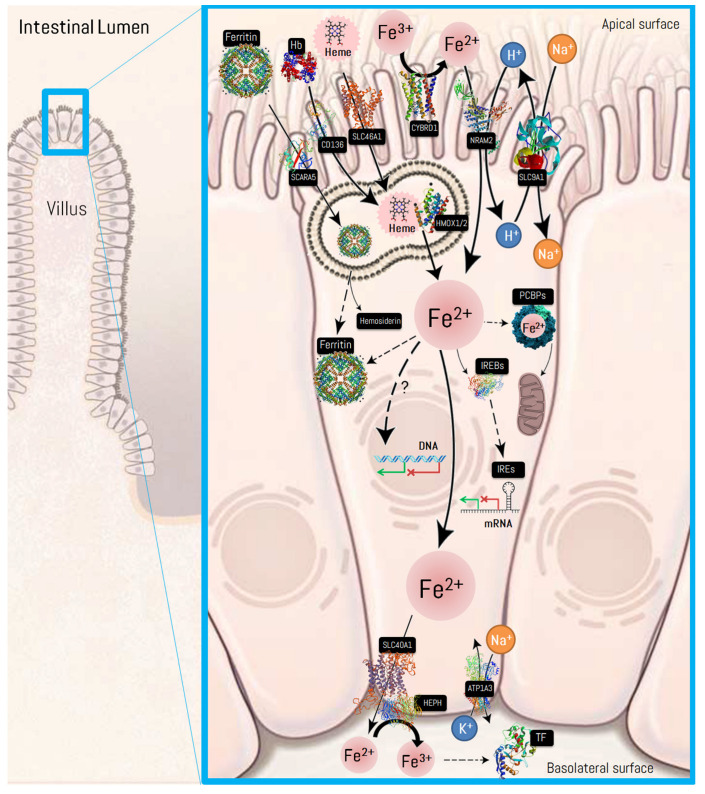
Iron absorption in the duodenum. The protein structures were taken from UniProtKB and PDB repositories. Structure prediction by homology modeling was carried out using SWISS-MODEL via the ExPASy web server if the protein structure was not available at UniProtKB or PKB. ATP1A3, basolateral sodium/potassium-transporting ATPase; CD163, scavenger receptor cysteine-rich type 1 protein M130; CYBRD1, cytochrome b reductase 1; CP, ceruloplasmin; Hb, hemoglobin; HEPH, hephaestin; HMOX1/2, heme oxygenases 1/2; IREBs, iron-responsive element-binding proteins; IREs, iron-responsive elements; mRNA, messenger RNA; NRAM2, natural resistance-associated macrophage protein 2; PCBP, poly(rC)-binding protein; SCARA5, scavenger receptor class A member 5; SLC9A1, apical sodium/hydrogen exchanger 1; SLC40A1, solute carrier family 40 member 1; SLC46A1, proton-coupled folate transporter; TF, transferrin. Source: designed by the authors (D.A.B.).

Interestingly, Fe is transported in the bloodstream bound to transferrin in its Fe^3+^ state. For this, the Fe^2+^ ion is oxidized by a ferroxidase Cu^2+^-dependent protein known as hephaestin (HEPH) at the basolateral surface of the duodenum [86,87], although, in most body cells, this process is achieved by the homolog ceruloplasmin (CP) [77,88]. The proton gradient that fuels several processes (e.g., Fe^2+^ uptake by NRAMP2 into enterocytes, or Fe^2+^ transport to the basolateral surface by SLC40A1) is maintained by the combined actions of apical sodium/hydrogen exchanger 1 (SLC9A1) and basolateral sodium/potassium-transporting ATPase (ATP1A3) antiporters. A representation of the processes mentioned above is shown in Figure 3. Thus, Fe transport and metabolism are regulated at different levels that involve multiple mechanisms. At the membrane, the regulation is mediated by plasma and organelle membranes, such as protein/solute carriers and the lysoendosomal trafficking membrane; cytosolic regulation involves the action of FTL/FHL1 and PCBPs; at the nucleus transcriptional (e.g., hypoxia-inducible factors, HIFs) and post-transcriptional (the IREB/IRE system) mechanisms are emphasized; however, this last seems to be the best-understood system [12,13,69]. The most relevant genes/proteins of Fe uptake and metabolism that were prioritized after manual curation are described in detail in Table 1.

## 4. Iron and the Immune System

**Figure 4:**
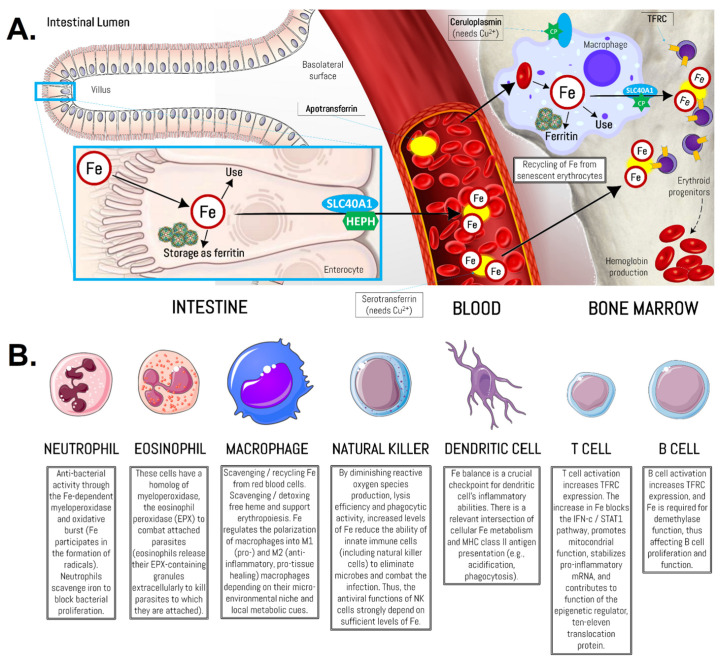
Integration of iron metabolism to hemoglobin production and immune cell function. (**A**) The figure represents the integration of Fe to hemoglobin through bone marrow macrophages. Blue ovals represent the SLC40A1 (also known as ferroportin). The green six-pointed star represents ceruloplasmin (CP), which needs Cu^2+^. Bright yellow ovals are pointed with arrows as apotransferrin (TF) while the orange bar on the membrane of erythroid progenitors (purple cells) represents the TFRC. (**B**) Fe bioavailability orchestrates complex metabolic programs in immune cell function and inflammation (for comprehensive reviews on this topic, please refer to [83,102,103,104]). Fe, iron; HEPH, hephaestin; SLC40A1, solute carrier family 40 members 1; TFRC, transferrin receptor protein 1. Source: designed by the authors (D.A.B.) using figure templates developed by Servier Medical Art (Les Laboratoires Servier, Suresnes, France), licensed under a Creative Common Attribution 3.0 Generic License. http://smart.servier.com/ (accessed on 20 February 2021).

In the last decades, scientific evidence has highlighted the close link between Fe and immune function [83]. Under physiological conditions, the immunocompetent cells capture the plasma circulating Fe^3+^ ion bound to transferrin through transferrin receptor protein 1 (TRFC, also known as CD71) [90], except for neutrophils, which are believed to lack this receptor [91]. However, the existence of TRFCs is associated with the grade of differentiation of the neutrophil lineage [92]. Similarly, resting T lymphocytes do not express TRFC [93] as they do not require Fe, but when lymphocyte activation occurs, the expression of the receptor is initiated in the G0 to G1 phase of the cell cycle. This ensures the presence of the necessary Fe for metabolic processes linked to the secretion of IL-2 [94], a cytokine with lymphoproliferative activity. A similar process occurs in natural killer (NK) cells, which do not express TRFC at rest and only do so after activation [95]. In these cells, Fe may influence the expression of the major histocompatibility complex (MHC) class I molecules that lead to NK cell activation [96]. Resting B lymphocytes express small amounts of TRFC [97], indicating reduced but constant transferrin requirements. After mitogenic stimulation, most B lymphocytes increase TRFC expression and, therefore, cellular Fe uptake [98]. Finally, resting macrophages exhibit TRFC expression and, in an environment rich in Fe, they increase the amount of these receptors to have Fe deposits, necessary in their phagocytic and cytotoxic activity (i.e., erythrophagocytosis) [98,99]. It is becoming clear that large differences in intracellular and extracellular Fe availability may affect macrophage functions [100]. It needs to be noted that erythroid precursors in the bone marrow take up transferrin-bound Fe from the blood. The level of transferrin-bound Fe in the blood is dynamic and is comprised of Fe absorbed from the diet by enterocytes, Fe stored in the liver and Fe released from splenic macrophages that recycle senescent erythrocytes [14,101] (Figure 4). 

In parallel, Fe^2+^ ion is a component of several metalloenzymes (forming the so-called iron-sulfur [Fe-S] clusters) [105,106]. These Fe-S clusters are indispensable for the viability of the cell (e.g., DNA maintenance, transcription/translation processes, and metabolic regulation) and might assemble not only in mitochondria but also in the cytosol and nucleus [107]. Furthermore, Fe is required in the formation of reactive oxygen species (ROS) [108,109] and, thereby, it is a critical component of key enzymes during the process of “respiratory burst” that occurs during phagocytosis [110,111]. Human lymphocytes produce several proteins crucial for regulating Fe levels, such as FTL/FTH1 and SLC40A1. For instance, the FTL/FTH1 complex acts as an Fe storage spherical shell (retaining it when there is too much in the body or releasing it when there is deficiency), while SLC40A1 is the “gateway” of Fe-containing cells (again releasing it or retaining it when necessary) [112,113]. The regulation of these proteins’ expression by lymphocytes plays an important role in Fe metabolism via the Fe-sensitive conformational equilibrium between cytosolic aconitase and the IREB/IRE system (which also needs Fe-S clusters) [114]. The FTH/FTL ratio is tissue and context-specific, and dynamically regulated, with FTH becoming more abundant during active inflammation [115]. 

The control of Fe levels (deficit or excess) is key to the immune system. Fe deficiency selectively prevents lymphocytic proliferation (specifically the Th1 subpopulation with less effect on Th2 lymphocytes), decreases the delayed hypersensitivity response, and modifies phagocytic activity [37]. Numerous Fe-containing metalloenzymes, such as myeloperoxidase of neutrophils and catalase, among others, are involved in bacterial destruction [83]. Specifically, regarding the innate response, it has been observed that people with insufficient Fe intake have less phagocytic capacity and a lower proportion of circulating neutrophils [116,117]. In normal conditions, the Fe present in the organism is superior to that required by microorganisms, but it is bound to proteins and is not available for bacterial growth, given that when there is a situation of excess Fe, bacterial proliferation is favored [118]. In addition, the association of low plasma Fe values with selective inhibition of Th1 proliferation has been described [119]. 

On the other hand, excess Fe is toxic to the body’s cells as it produces peroxidation of cell membranes and intracellular organelles. In addition, an overload of Fe generates immunosuppression as it decreases the proliferative capacity of Th and Tc lymphocytes, and increases the activity of Treg lymphocytes; it has also been established that elevated plasma Fe values interfere with the production of the cytokine interferon-gamma (IFN-γ) [120]. With respect to the adaptive response, the low Fe intake induces a decrease in proliferative capacity by interference in the translocation and activation of protein kinase C (PKC), a decrease of secondary messengers, such as PIP2, and lower capacity of production of cytokines, such as TNFα and IL-2 [112,121]. This scenario currently relates Fe to humoral immunity since this micronutrient is required for B-cell proliferation and the production of antibodies to antigens [122].

Considering the processes mentioned above, it is important to highlight the role of HAMP, a key liver-derived protein, in regulating the body Fe levels, which is also synthesized by the lymphocytes [123,124]. HAMP is highly conserved from zebrafish to humans [125] and was initially described as a cysteine-rich antimicrobial peptide [126,127]. Notwithstanding this, experiments with murine model hepatocytes treated with Fe overload (carbonyl iron and iron-dextran), or knockout for β2-microglobulin, showed increases in HAMP expression [128]; therefore, it is considered the main circulating regulator of Fe absorption and distribution across tissues. Fe-sensing proteins, such as TRFC, homeostatic iron regulator (HFE), and hemojuvelin, may stimulate bone morphogenetic proteins (BMPs) that are able to act on the SMAD4 pathway, which is the critical regulator of *HAMP* expression [129,130]. Since BMPs are a subset of the transforming growth factor-beta (TGF-β) superfamily, the cytokine-mediated activation of SMAD4 and JAK/STAT3 pathways (e.g., via the pro-inflammatory action of IL-6) also regulates HAMP expression [131]. Mechanistically, HAMP induces the internalization of SLC40A1 (ferroportin) by binding to it, which modulates Fe release from the cells [132]. In this regard, HAMP has also been shown to occlude ferroportin [133]. In general, HAMP also affects lymphocyte activation, suggesting that the regulation of intraleucocyte Fe and the immune response is highly related [67]. 

Consequently, the role of Fe on innate and adaptive immune functions, whether due to its participation in antioxidant mechanisms or not, has also been demonstrated by the decrease in certain responses in an Fe-deficient population [134,135]. In the context of bacterial infection, HAMP reduces Fe availability in the blood and, thus, helps to control the infection, since bacteria need Fe to divide [136,137]. However, HAMP-induced hypoferremia might be detrimental to cellular defense against viral infections, although the mechanism underlying this correlation is unclear [137]. This battle for Fe between the human and the virus-mediated physiological effects is of current interest due to the low extracellular Fe availability consistently reported in severe COVID-19—it seems HAMP might play a substantial role as a contributing factor to a worsening clinical course [138].

## 5. Identification of Potential Biomarkers of Stress-Induced Immunosuppression

To ensure survival and optimal activity of the human body, the immune function may be enhanced in response to short-term transient stress (lasting minutes to hours), but it tends to diminish if the stressor continues over days to months (immunosuppression) [29]. Aware of the important differences that account for specific responses after a given stress condition, here we used the physiological stress generated by physical exercise as a model to analyze the stress-induced immunosuppression. The stress magnitude of a given physical exertion will depend on its intensity and duration (allostatic challenge) [18]. It is worth noting that a high physical activity level is associated with a reduction in the allostatic load [27,35]; hence, a programmed and appropriate dose of physical exercise will improve immunological health across the lifespan [139], and limits or delays immunosenescence and low-grade inflammation in older adults [140]. Based on exercise dose, studies have shown: (i) an enhanced immunosurveillance after acute exercise (<60 min); (ii) a transient immune dysfunction after intensive workloads and prolonged exercise, and (iii) a higher illness risk after heavy/strenuous and prolonged exercise workloads [141,142]. 

Within the framework of stimulus-response theory and the allostasis model [26], one of the aims of biomedical research is to evaluate and identify sensitive biomarkers that reflect physiological derangements across the stress response [27]. As shown in Figure 1, biological stressors produce cytokine-mediated inflammation followed by a rise in T-cell activity (especially Th1 within hours and days) and, finally, an elevation of B-cell activity (Th2 response after days and weeks) [29]. Since exercise of different intensities has diverse effects on cell-mediated immunity, regulated by a complex mechanism in connection to inflammation [142], recent studies have assessed alterations in the innate immune system to identify easy-to-measure biomarkers of exercise-induced immunosuppression [143,144]. In accordance with the large interactions between the immune system and Fe status in athletes, some studies have shown that HAMP [49], phagocytic activity [145], and TF [143] could be used as biomarkers of exercise-induced immunosuppression. Nevertheless, the detailed mechanisms behind the observed changes in Fe-related proteins associated with exercise-induced alterations in the immune function are still unknown, and other biological markers in the different phases of its allostatic response are needed. Hence, bioinformatics might provide important information on system-level cellular processes and future directions for experimental research in exercise immunology [146].

**Figure 5:**
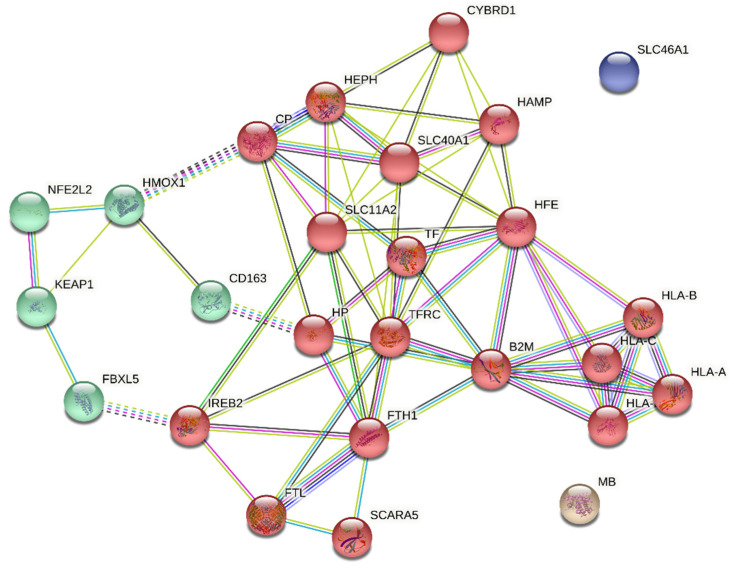
Protein-protein interactions network of iron metabolism and the immune system. The colored nodes represent the results of the Markov cluster algorithm to group proteins in two main biological functions: presentation of peptide antigens to the immune system (red) and ubiquitination, endocytosis, and degradation processes of proteins related to Fe metabolism in immune cells (e.g., macrophages) (green). The colors of interactions correspond to: known from curated databases (cyan), experimentally determined (purple); predicted interactions based on gene neighborhood (green), gene fusions (red), and gene co-occurrence (dark blue); and others, such as text-mining (yellow), co-expression (black), and protein homology (light blue). The input proteins were: CD163, scavenger receptor cysteine-rich type 1 protein M130; CP, ceruloplasmin; CYBRD1, cytochrome b reductase 1; FTH1, ferritin heavy chain; FTL, ferritin light chain; HAMP, hepcidin; HEPH, hephaestin; HMOX1, heme oxygenase 1; IREB2, iron-responsive element-binding protein 2; MB, myoglobin; SCARA5, scavenger receptor class A member 5; SLC11A2, natural resistance-associated macrophage protein 2; SLC40A1, solute carrier family 40 member 1; SLC46A1, proton-coupled folate transporter; TF, transferrin; TFRC, transferrin receptor protein 1. The network is available at https://version-11-0b.string-db.org/cgi/network?networkId=b1HF4feAW2Nr (accessed on 17 June 2021).

Based on this, we built a PPIN of the prioritized proteins of Fe metabolism by mapping them into the STRING tool to evaluate the potential interactors directly related to the immune system (Figure 5). The preliminary topological analysis of the network showed an average local clustering coefficient of 0.535 with an average node degree equal to 5.15. The main connected component was constructed with 24 nodes and 67 edges (MB and SLC46A1 did not connect to other proteins under the settings of this network, but both interact with members of the human leukocyte antigen system). The very low protein-protein interactions enrichment *p*-value (<1.0^−16^) indicated that the nodes were not random and that the observed number of edges was significant, and this was expected considering all input proteins belong to Fe metabolism.

Based on the GO annotation, the Kyoto Encyclopedia of Genes and Genomes (KEGG) pathway analysis, and the Protein Families (PFAM) Protein Domains analysis, an enrichment analysis of the network was performed (Table 2). Besides Fe transport and oxidoreductase activity, the GO molecular function showed that peptide antigen binding and antigen peptide transporter 1 (TAP binding) are among the top functions of the network. The GO cellular component revealed that most proteins are located in the recycling endosome, MHC class I protein complex, early endosome, cell surface, and the HFE-transferrin receptor complex. As expected, the KEGG pathway enrichment analysis showed that these proteins were mainly associated with mineral absorption and ferroptosis; however, the PPIN of prioritized proteins of Fe metabolism are highly involved in pathways modulating the antigen processing and presentation, allograft rejection (a consequence of the recipient’s alloimmune response to non-self-antigens expressed by donor tissues), and graft-versus-host disease. Interestingly, the PFAM analysis showed conservation of the immunoglobulin C1-set domain, the MHC_I C-terminus, and the MHC class I alpha chain, alpha1 alpha2 domains. Furthermore, clustering the network with a Markov algorithm allowed identifying that several proteins were grouped in two main biological functions: (i) presentation of peptide antigens to the immune system with the involvement of redox reactions of Fe, heme, and Fe trafficking/transport; and (ii) ubiquitination, endocytosis and degradation processes of proteins related to Fe metabolism in immune cells (e.g., macrophages). 

**Figure 6:**
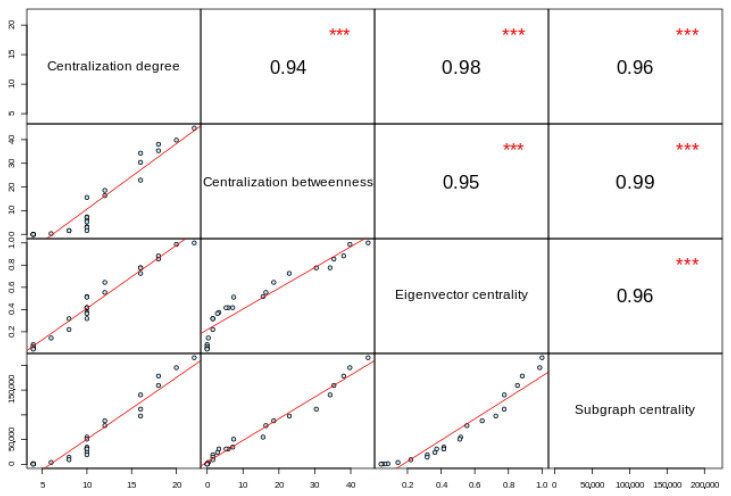
Matrix-like plot showing pairwise correlations of the centrality scores. The upper-right part shows the numerical correlation between the given topological features, whereas the lower-right part of the matrix is the scatterplot of one feature against another. These high correlations between centrality metrics provide useful insights into the potential of different nodes within a network [147]; particularly, the presence of highly connected nodes is likely to be rated as central by other metrics, representing a putative core that for the aims of this study might result in potential biomarkers. Figures were obtained from the Network Analysis Profiler v2.0 [61]. *** Statistically significant correlation (*p* < 0.001).

We explored the topological features of the network and ranked the nodes based on centrality measures. Table 3 shows the top-ranked proteins with HFE, TFRC, beta-2 microglobulin (B2M), and SLC11A2 as the nodes with higher scores. A matrix-like plot showing pairwise comparisons shows the high correlation between any combination of selected intra-network topological features (Figure 6) [61].

### 5.1. Evidence-Based Verification of the Identified Potential Biomarkers

We highlight that our results are meaningful since almost all the identified potential biomarkers agree with available experimental evidence and are currently part of several immunological/biomarkers databases. In contrast, the others are emerging genetic markers for different stress conditions, including exercise (Table 4). It is accepted that markers of Fe status not only determine the cardiorespiratory fitness but also should be interpreted in the context of the individuals’ stimuli-response process (e.g., competition season, recent training intensity, frequency, duration, inflammation state, and nutritional changes) [148]. We must point to the scientific community’s current consensus that establishes the need to assess Fe levels, HAMP, total Fe-binding capacity, TF saturation, soluble TFRC and FTH1/FTL (ferritin) to monitor the Fe metabolism-related exercise-induced physiological perturbations in recreational and elite athletes [41,49,149]. 

HFE was identified as the top biological regulator after our network topology analysis. The *HFE* gene encodes this MHC-class I type membrane protein [150]. HFE binds to B2M and the extracellular domain of the TFRC to regulate HAMP expression and, thus, the closed link of the immune function and Fe metabolism [151]. A high prevalence of two *HFE* mutations is present in professional endurance athletes (49.2%) compared with sedentary controls (33.5%): C282Y (rs1800562) and H63D (rs1799945) [149]. It has been reported that subjects bearing the H63D polymorphism have lower cardiovascular fitness and achieve lower maximal power output than a control group even in the absence of Fe accumulation (no differences were seen in blood FTH1/FTL concentrations) [152]. Intriguingly, physical exercise with increasing intensity over time seems to take a distinct HAMP pathway depending on the modulating effect of the HFE genotype, given that young male H63D carriers normally present higher basal HAMP concentrations than wild-type males [153]. 

It should be noted that only 63.6% of amateur endurance runners harboring the H63D polymorphism have shown an increase in HAMP levels after a marathon (mean race time: 3 h 44 min 35 s) [154]. Conversely, Kortas et al. (2020) demonstrated that a reduction in body Fe stores might constitute an important aspect of the health-promoting effect of exercise, regardless of the HFE genetic background in non-physically active older women [155]. In a recent meta-analysis, Semenova et al. (2020) concluded that the *HFE* H63D polymorphism is strongly associated with elite endurance athlete status (association between the *HFE* G allele and high VO_2max_ in male athletes was reported) regardless of ethnicity and cardiorespiratory capacity [156]. Considering that these two *HFE* polymorphisms (C282Y and H63D) can be used to predict the risk of hereditary hemochromatosis, Thakkar et al. (2021) classified athletes based on low risk or medium/high risk using an algorithm that integrated the *HFE* genotype. They reported that individuals with the medium- or high-risk genotype were ~8% faster and showed a ~17% higher VO_2peak_ than those with the low-risk genotype [157]. 

Interestingly, independent of age, carriers of either C282Y and/or H63D polymorphisms have shown a higher load of Fe in the putamen (a component of the dorsal striatum in the brain), higher TF saturation, and lower TF and TFRC in blood than non-carriers; furthermore, the putaminal Fe level positively correlated with cognitive and motor function [158]. According to the authors, *HFE* status is characterized by higher regional brain Fe load across adulthood and is linked to cognitive and motor function in healthy adults. In summary, (i) these two single nucleotide polymorphisms of *HFE* (C282Y and H63D) can be combined to categorize individuals as having a high, medium, or low risk for Fe overload; (ii) while the genetic risk for iron overload may have a favorable impact on performance, it is necessary for athletes with a medium or high risk to avoid Fe supplementation as this could lead to adverse health outcomes and diminished performance [159].

TFRC and FTH1 (part of the FTH1/FTL complex or ferritin) were other important identified proteins. Although TFRC is a membrane protein, a truncated soluble form, known as soluble TFRC, correlates with the cellular expression at the membrane and rises with Fe needs [14]. Soluble TFRC is elevated in acute states and constitutes a marker of Fe deficiency in tissues rather than a measure of anemia [160]. It is worth mentioning that highly trained (athletes) [161] and untrained [162] healthy individuals exhibit increased soluble TRFC levels solely in response to high-intense or maximal exercise with a subsequent return to baseline during the recovery period. On the other hand, FTH1/FTL might be slightly altered after energy- or mechanical stress, including exercise and nutrition interventions [163]. Indeed, a recent meta-analysis has shown that blood FTH1/FTL increases significantly after intensified non-resistance-based training [164]. Since FTH1/FTL (ferritin) and TF (transferrin) are not sufficiently accurate, as they are both elevated in any anemia or inflammation process [160], the ferritin index (ratio of soluble TFRC to log ferritin) has been suggested as a more stable, reliable and sensitive marker [165]. 

Interestingly, this index has a lower mean day-to-day variability and remains stable, despite daily changes in FTH1/FTL or soluble TFRC levels; and has also been used to evaluate the effect of different exercise training phases on whole body Fe in endurance athletes [166]. Sierra et al. (2019) have reported that both TF concentration and saturation increase immediately after prolonged exercise-induced stress (São Paulo International Marathon) and reduce up to 15 days after that; in addition, the authors found that the *ACTN3* R577X polymorphism might partially explain the different hematological responses in endurance athletes given that individuals bearing the RR genotype seem to be more susceptible to hemolytic anemia and hematuria [167]. This highlights the relevance of monitoring Fe supplementation and renal function evaluation on a genotype-dependent basis, as mentioned previously. 

We also found HAMP within the list of identified potential biomarkers, which agrees with current practice to monitor athletes [168]. Besides increasing FTH1/FTL, augmentation in HAMP levels has been reported after seven days of high-training load in elite male rowers [166]. As mentioned previously, HAMP increases in response to inflammation [169,170] and is an important regulator of Fe status in several physiological conditions (e.g., hemolysis, hematuria, and intestinal bleeding) [170,171]. HAMP concentrations are normally expressed in nanomoles per liter (1 nM serum HAMP equals 2.79 μg·L^−1^) [172]. It should be noted that an increase in IL-6 levels has been linked to the enhanced expression of *HAMP* in the liver [129]. Experimental evidence in animal models has demonstrated increases in plasma IL-6 concentration, which correlated with liver expression of the IL-6 alpha receptor (*IL6R*) and suppressor of cytokine signaling 3 (*SOCS3*) after intense exercise [173]. Similarly, Liu et al. [174] reported exercise-induced anemia in rats and found IL-6 concentrations induced hepatic HAMP expression. These significant increases in IL-6 and HAMP have been demonstrated in young females after acute exercise bouts (60 and 120 min at 65% of VO_2max_) [175], although Fe levels seem not to be affected after a period (until four weeks) of high load in young athletes [166]. It must be highlighted that H63D HFE gene polymorphism has a modulating impact on HAMP secretion [153]. 

Two of the identified potential biomarkers have emerged. SLC11A2 (DMT1) and SLC40A1 (ferroportin) have been studied as potential molecular regulators of neuroinflammation [176] and exercise-induced changes in Fe status [177]. For example, inflammation in aging and neurodegenerative phenotypes is associated with Fe accumulation in the central nervous system through the altered expressions of SLC11A2, SLC40A1, and HAMP [178]. Neuroinflammation has led to overexpression of SLC11A2 in neurons, astrocytes, and microglia and a parallel reduction in SLC40A1 expression [176]. Choi et al. (2021) showed that treadmill exercise reduced intracellular Fe accumulation, probably by decreasing TF, TFRC, and SLC11A2 (lower Fe transport into cells) while increasing SLC40A1 expression (Fe-releasing protein) in the motor cortex of aging Alzheimer’s disease mice [179]. 

Furthermore, while no changes were seen in the sedentary and the strenuously exercised groups, it seems that moderate-intensity exercise in healthy animal models increases the expression of SLC11A2 with IRE and SLC40A1, but down-regulates HAMP, which might have improved Fe duodenal reabsorption (higher Fe status) [180]. Contrariwise, strenuously exercised rats have shown under-expression of duodenal SLC11A2, heme-carrier protein 1, and SLC40A1, which may partially explain the reduced Fe absorption stress-associated stress anemia after intensive exercise [174]. Interestingly, recent research performed by Wuyun et al. (2021) found that a *SLC11A2* 258/258 bp homozygous genotype and 258 bp alleles are overrepresented in elite Chinese long-distance runners and concluded that this might be considered a genetic marker due to a significant association with cardiorespiratory fitness [181]. A previous genetics case study has also associated a mutation in *SLC11A2* with a slight increase in serum Fe level in severe anemia and the hepatic Fe overload phenotype [182]. In addition, the *SLC40A1* R178Q mutation (rs1449300685), among other variants [183], has recently been shown to affect the HAMP-SLC40A1 interaction, which might contribute to the spectrum of Fe overload [184,185]. Therefore, the different magnitude and direction in the expression of SLC11A2 and SLC40A1 might depend on the allostatic load of the biological system and the basal immune function (e.g., genetics—see Figure 1), but further research is warranted to establish accurate mechanisms.

Future studies might evaluate the validity and sensitivity of other identified proteins that are less monitored biomarkers in the exercise and sports fields, such as B2M, HEPH, and CP. Serum levels of B2M are normally elevated in human immunodeficiency virus infection and acquired immunodeficiency syndrome, rheumatoid arthritis, and hematologic malignancies—which possibly retard the generation of monocyte-derived dendritic cells and might be involved in the down-regulation of major histocompatibility complex class I molecules, inactivation of Raf/MEK/ERK cascade and NF-κB, and activation of STAT3 [186]. Exercise in young hypertensive patients produced a decrease in B2M, although no changes were seen in healthy control individuals [187], which might contribute to the health benefits that have been seen after a moderate-intensity exercise program in hypertensive postmenopausal women [188]. B2M has been described as a conservative multifunctional regulator of immune surveillance and modulation of immune function [189]. B2M has not only been reported as a classic marker to assess kidney function [190] but also as an emerging screening tool in several non-renal diseases, such as peripheral arterial disease [191], cancer [192], and aging-related oxidative stress [193]. Both plasma and urinary B2M levels may be reliably and cost-effectively measured [194]. Although it has also been used as a housekeeping or reference gene in exercise and nutrition interventions [195,196], researchers should consider that B2M might be unsuitable for some conditions [197,198]. It has been demonstrated that reduction in the intestinal HEPH and CP-ferroxidase activity may impair Fe absorption and Fe release from intracellular stores, respectively, which decreases Fe levels and results in disturbances of Fe delivery in the bone marrow to support erythropoiesis [199]. In the absence of HEPH, there is anemia, possibly due to Fe malabsorption to the systemic circulation [69]. In addition, downregulation of HEPH expression has been found during the extrahepatic [200] and intrahepatic [201] acute-phase response in immunosuppressant-induced animal models. Importantly, similar to our results, a recent bioinformatics analysis based on mRNA expression data indicated HEPH as a potential novel prognostic biomarker for lung cancer pathologies [202]. More research is necessary to evaluate these markers.

### 5.2. Limitations, Strengths, and Future Directions

The results of this study should be discussed in light of the following limitations and strengths. Firstly, databases for reviewing literature were restricted to PubMed, ScienceDirect, and Google Scholar. Secondly, the bioinformatics-assisted review is based on the FAIR guiding principles [203] and, thereby, takes advantage of (i) the scientific soundness of manual curation, and (ii) the use of simple, freely accessible, and curated bioinformatics tools to enhance cross-referencing, enrich the biological interpretation and annotation of molecular entities. A bioinformatics-assisted approach for reviewing literature exceeds human-based or machine-based individual methods in terms of effectiveness due to a refined retrieval and curation process. Thirdly, it should be considered that the bioinformatics enrichment analysis and the conclusions from non-clinical research should be interpreted with caution, given that they might not fully reflect adaptive responses in humans during changes in immune function after a given stress response. Fourthly, we have used a mechanistic-based approach to evaluate cellular and systemic changes in Fe metabolism. However, experimental research is still needed to better comprehend the molecular and cellular mechanisms that might link the bidirectional alterations in Fe metabolism and immune system. Fifthly, we limited our discussions to exercise as a model example of stress-induced changes in immune function and, thereby, invite readers and researchers to explore other phenotypes, such as cancer [204], neuroinflammatory diseases [205], and infections [138,206], among others. Finally, we must highlight the high performance of our bioinformatics-assisted approach to identify potential molecular and genetic biomarkers based on molecular prioritization, enrichment, and network topology analysis. This has been successfully implemented to identify proteins/genes that might have important biological functions [207] and biomarkers [208,209]. Our evidence-based verification closely matched our identified biological markers; nonetheless, we are aware that more research is needed to validate the proposed biomarkers in different stages during the acute and chronic inflammatory response and how this may affect human adaptation processes. 

We expect that this study’s results might contribute to hypothesis generation for subsequent research to decipher the mechanisms that link several Fe metabolism-related genes/proteins and the immune response in several phenotypes. Researchers should take advantage of the different wet- and dry-based immunosuppression models to study: (i) the novel findings regarding the intrahepatic acute-phase response-like reaction and Fe overload [201]; (ii) the large inter-individual variability in biomarker responses that might result from genetic-derived individual responses, such as the variants in *HFE*, *SLC11A2*, *SLC40A1* and *HAMP*; (iii) sex-based differences, given that pre-clinical research has shown, for example, higher levels of ferroportin protein or reduction of hepatic HAMP mRNA in the liver, spleen, and kidney in males than in females [210]—for a recent and comprehensive review of the current knowledge in regards to Fe status and Fe supplementation for the female athlete, please refer to [211,212]); (iv) altitude-based differences, its effects on Fe regulation and the impact on the immune function [213]; (v) the potential of computational prediction by PPI mapping to help determine target proteins/genes considering that the functional study of biomarkers is a time- and cost-consuming process [214], especially under the immunoinformatics paradigm which opens a new door into the study of the immune response in different biological contexts [215].

## 6. Conclusions

Fe is an important micronutrient that may constitute a double-edged sword since the two extremes of nutritional status (i.e., Fe deficiency or overload) have harmful effects on innate and acquired immunity. Thus, Fe is highly regulated at different cell levels, including membrane (e.g., protein/solute carriers and lysoendosomal trafficking), cytosolic (e.g., FTL/FTH1 complex, Fe-chaperones), transcriptional, and post-transcriptional (i.e., IREB/IRE system) levels. Furthermore, large differences in intracellular and extracellular Fe levels may affect the immune response; in fact, emerging evidence also refers to critical phenotypes based on intrahepatic or extrahepatic concentrations of Fe metabolism-related proteins. In this sense, our bioinformatics- and network topology-based analysis identified potential molecular biomarkers related to the close link between Fe metabolism and immune function. In detail, the graph-based Markov algorithm grouped several prioritized proteins in two main clusters: (i) presentation of peptide antigens to the immune system with the involvement of redox reactions of Fe, heme, and Fe trafficking/transport; and (ii) ubiquitination, endocytosis, and degradation processes of proteins related to Fe metabolism in immune cells (e.g., macrophages).

Importantly, the novelty of the approach and our results were meaningful since the identified potential biomarkers were in agreement with the current experimental evidence, belong to several immunological/biomarkers databases, and/or are emerging genetic markers for different stressful conditions. This highlights the high efficiency of human-machine collaboration, using human-generated feedback to improve computer results. Besides the response to exercise, the evaluation of molecular mechanisms and the clinical implications of Fe level (deficiency or overload) are important research areas for the design and implementation of nutrition- or exercise-based immunomodulatory interventions in different contexts (e.g., obesity, cancer, neuroinflammatory diseases, infections). There is no doubt that the identified biomarkers deserve further research to confirm effects and derive clinical recommendations; thus, we encourage researchers to use the information contained in this study and adopt a more intuitive, integrative, and allostatic view based on complex systems, network analysis, and the ever-changing and adaptive responses of biological organisms (a ‘Bio-Logic’ approach). 

## Figures and Tables

**Table 1:** Characteristics of prioritized proteins of iron metabolism.

Recommended Name (Alternative Names)	Gene Name (Location)	Ensembl ID	Protein Features (UniProtKB/PDB Entry)	Cellular Location	Molecular Function	Protein Expression * (BioGPS ID)
Cytochrome b reductase 1 (Duodenal cytochrome b; Ferric-chelate reductase 3)	*CYBRD1* (2q31.1)	ENSG00000071967	Length: 286 Mass: 31,641 Da (Q53TN4/5ZLE)	Integral component of membrane. Present at the brush border of duodenal enterocytes where it probably reduces dietary Fe^3+^ thereby facilitating its transport into the mucosal cells.	Ferric-chelate reductase that reduces Fe^3+^ to Fe^2+^. Uses ascorbate as electron donor. May be involved in extracellular ascorbate recycling in erythrocyte membranes. May also act as a ferrireductase in airway epithelial cells.	Thyroid gland, small intestine, colon, testis, gallbladder, ovary, breast endometrium (79901)
Natural resistance- associated macrophage protein 2—NRAM2 (Solute carrier family 11 member 2; Divalent metal ion transporter 1 [DMT1])	*SLC11A2* (12q13.12)	ENSG00000110911	Length: 568 Mass: 62,266 Da (P49281/5F0L)	Integral component of plasma membrane. Present at the apical plasma membrane where it is involved in Fe uptake into duodenal enterocytes. May serve to import Fe into the mitochondria.	Important in metal transport, in particular Fe. Can also transport manganese, cobalt, cadmium, nickel, vanadium and lead. May play an important role in hepatic Fe accumulation and tissue Fe distribution.	Salivary gland, cerebral cortex, adrenal gland, bronchus, lung, stomach, colon, rectum, liver, gallbladder, pancreas, kidney (4891)
Proton-coupled folate transporter (Heme carrier protein 1)	*SLC46A1* (17q11.2)	ENSG00000076351	Length: 459 Mass: 49,771 Da (Q96NT5/-)	Apical plasma membrane. Localizes to the apical membrane of intestinal cells in Fe-deficient cells, while it resides in the cytoplasm in Fe-replete cells.	It has been shown to act both as an intestinal proton-coupled high-affinity folate transporter and as an intestinal heme transporter, which mediates heme uptake from the gut lumen into duodenal epithelial cells.	Testis, small intestine, duodenum, colon (113235)
Scavenger receptor cysteine-rich type 1 protein † (Hemoglobin scavenger receptor)	*CD163* (12p13.31)	ENSG00000177575	Length: 1156 Mass: 125,45 Da (Q86VB7/-SWISS-MODEL Repository Q86VB7)	Extracellular region or secreted and plasma membrane. Acute phase-regulated receptor involved in clearance and endocytosis of hemoglobin/haptoglobin complexes.	May play a role in the uptake and recycling of Fe, via endocytosis of hemoglobin/haptoglobin and subsequent breakdown of heme. Binds hemoglobin/haptoglobin complexes in a calcium-dependent and pH-dependent manner.	Lung, spleen, bone marrow, lymph node, appendix, tonsil (9332)
Heme oxygenase 1 —HMOX-1	*HMOX1* (22q12.3)	ENSG00000100292	Length: 288 Mass: 32,219 Da (P09601/1N3U)	Endoplasmic reticulum membrane and perinuclear region of cytoplasm. Under physiological conditions, the activity of heme oxygenase is highest in the spleen, where senescent erythrocytes are sequestrated and destroyed.	Heme oxygenase cleaves the heme ring at the alpha methene bridge to form biliverdin. Biliverdin is subsequently converted to bilirubin by biliverdin reductase.	Lung, duodenum, small intestine, spleen, bone marrow, placenta, appendix, lymph node, tonsil (3162)
Ferritin heavy chain	*FTH1* (11q12.3)	ENSG00000167996	Length: 183 Mass: 21,226 Da (P02794/1FHA)	Cytosol, extracellular exosome, autolysosome, protoplasm.	Stores Fe in a soluble, non-toxic, readily available form. Has ferroxidase activity. Fe is taken up in the ferrous form and deposited as ferric hydroxides after oxidation. Also plays a role in delivery of Fe to cells.	Cerebral cortex, bone marrow, hippocampus, small intestine (2495)
Ferritin light chain	*FTL* (19q13.33)	ENSG00000087086	Length: 175 Mass: 20,020 Da (P02792/2FFX)			Cerebral cortex, cerebellum, lung, liver, kidney (2512)
Scavenger receptor class A member 5— (Ferritin receptor) †	*SCARA5* (8p21.1)	ENSG00000168079	Length: 495 Mass: 53,994 Da (Q6ZMJ2/- SWISS-MODEL Repository Q6ZMJ2)	Integral component of plasma membrane.	Ferritin receptor that mediates non-transferrin-dependent delivery of Fe. Mediates cellular uptake of ferritin-bound Fe by stimulating ferritin endocytosis from the cell surface with consequent Fe delivery within the cell.	Adrenal gland, stomach, small intestine, colon, rectum, tonsil, gallbladder, lymph node, (286133)
Poly (rC)–binding proteins	*PCBP2* (12q13.13)	ENSG00000197111	Length: 365 Mass: 38,580 Da (Q15366/2AXY)	Cytosol, extracellular region or secreted, nucleus and other cell locations.	As a chaperone, promotes intracellular Fe flux. It can directly receive Fe^2+^ from CYBRD1 or transfer Fe to the Fe^2+^ exporter, SLC40A1.	Cerebellum, bronchus, oral mucosa, stomach, liver, testis, kidney (5094)
Solute carrier family 40 member 1 (Ferroportin 1)	*SLC40A1* (2q32.2)	ENSG00000138449	Length: 571 Mass: 62,542 Da (Q9NP59/-)	Basolateral plasma membrane and integral component of plasma membrane.	May be involved in Fe export from duodenal epithelial cell and in transfer of Fe between maternal and fetal circulation. Mediates Fe efflux in the presence of a ferroxidase (hephaestin and/or ceruloplasmin).	Bone marrow, duodenum, small intestine, smooth muscle, skeletal muscle (30061)
Hephaestin	*HEPH* (Xq12)	ENSG00000089472	Length: 1158 Mass: 130,44 Da (Q9BQS7/- SWISS-MODEL Repository Q9BQS7)	Basolateral plasma membrane and integral component of plasma membrane.	May function as a ferroxidase for Fe^2+^ to Fe^3+^ conversion and may be involved in copper transport and metabolism. Implicated in [Fe] regulation and may mediate Fe efflux associated with SLC40A1.	Cerebral cortex, hippocampus, thyroid gland, lungs, stomach, small intestine, liver, pancreas, (9843)
Ceruloplasmin	*CP* (3q24-q25.1)	ENSG00000047457	Length: 1065 Mass: 122,20 Da (P00450/1KCW)	Extracellular region or secreted, plasma membrane, endoplasmic reticulum lumen and lysosomal membrane.	Ceruloplasmin is a blue, copper-binding (6–7 atoms per molecule) glycoprotein. It has ferroxidase activity oxidizing Fe^2+^ to Fe^3+^ without releasing radical oxygen species. It is involved in Fe transport across the cell membrane.	The RNA is highly expressed in the liver. Mainly found in the bloodstream (1356)
Transferrin	*TF* (3q22.1)	ENSG00000091513	Length: 698 Mass: 77,064 Da (P02787/1A8E)	Secreted. Blood microparticle, extracellular exosome, and extrinsic component of external side of plasma membrane.	Transferrins are Fe-binding transport proteins that can bind two Fe^3+^ ions in association with the binding of an anion, usually bicarbonate. They are responsible for the transport of Fe from sites of absorption and heme degradation to those of storage and utilization.	The RNA is highly expressed in the liver. Protein is highly expressed in placenta and testis, although is mainly found in the bloodstream (7018)
Transferrin receptor protein 1	*TFRC* (3q29)	ENSG00000072274	Length: 760 Mass: 84,871 Da (P02786/1CX8)	Integral component of plasma membrane, endosome (clathrin-coated vesicle membrane), blood microparticle. Positively regulates T and B cell proliferation through Fe uptake.	Cellular uptake of Fe occurs via receptor-mediated endocytosis of ligand-occupied transferrin receptor into specialized endosomes. Endosomal acidification leads to Fe release. The apotransferrin-receptor complex is then recycled to the cell surface with a return to neutral pH and the concomitant loss of affinity of apotransferrin for its receptor.	Placenta, bone marrow, cerebellum, hippocampus, adrenal gland, bronchus, lung, oral mucosa, esophagus, duodenum, colon, rectum, urinary bladder, testis (7037)
Hepcidin	*HAMP* (19q13.12)	ENSG00000105697	Length: 84 Mass: 9408 Da (P81172/1M4E)	Extracellular region or secreted. Controls the major flows of Fe into plasma: absorption of dietary Fe in the intestine, recycling of Fe by macrophages, which phagocytose old erythrocytes and other cells, and mobilization of stored Fe from hepatocytes.	Liver-produced hormone that constitutes the main circulating regulator of Fe absorption and distribution across tissues. Acts by promoting endocytosis and degradation of SLC40A1 (ferroportin), leading to the retention of Fe in Fe-exporting cells and decreased flow of Fe into plasma.	The RNA is highly expressed in the liver although very low expression levels are found in heart muscle and spinal cord. Mainly found in the bloodstream. (57817)
Iron-responsive element-binding protein 2 † (Iron regulatory protein 2)	*IREB2* (15q25.1)	ENSG00000136381	Length: 963 Mass: 105,05 Da (P48200/ SWISS-MODEL Repository P48200)	Cytoplasm, mitochondrion.	RNA-binding protein that binds to iron-responsive elements (IRES), which are stem-loop structures found in the 5′-UTR of ferritin, and delta aminolevulinic acid synthase mRNAs, and in the 3′-UTR of transferrin receptor mRNA.	Cerebellum, parathyroid gland, adrenal gland, oral mucosa, stomach, small intestine, kidney, prostate (3658)

Data were extracted from several bioinformatics databases/repositories (Ensembl, UniProtKB, PDB, Gene Ontology, BioGPS, and the Gene Expression Atlas). We strongly advise that the scientific community visit these databases to check the recommended names of these genes/proteins to standardize their use. Use the cross-reference to BioGrid, IntAct, KEGG, MINT, or other databases to analyze protein-protein interactions, metabolic networks, and signaling pathways. Many other bioinformatics tools are currently available. For biological process analysis, please see the functional enrichment of the protein-protein interaction network below. † The protein structure was not available in either UniProtKB or PDB repositories; therefore, a structure prediction by homology-modeling was carried out using SWISS-MODEL via the ExPASy web server [89]; * We reported the tissues with high and/or medium expression scores obtained from the Gene Expression Atlas. For more details regarding expression in different tissues or conditions (pathologies), visit Gene Expression Atlas using the gene name or BioGPS with the corresponding ID number.

**Table 2:** Results of the functional enrichment analysis of the PPIN.

Biological Process (GO)		
GO-term	Description	FDR *p*-value
GO:0055072	iron ion homeostasis	9.58 × 10^−31^
GO:0006879	cellular iron ion homeostasis	5.91 × 10^−30^
GO:0006826	iron ion transport	3.30 × 10^−20^
GO:0000041	transition metal ion transport	7.82 × 10^−20^
GO:0019725	cellular homeostasis	6.48 × 10^−16^
**Molecular Function (GO)**		
GO-term	Description	FDR *p*-value
GO:0005381	iron ion transmembrane transporter activity	5.37 × 10^−7^
GO:0016722	oxidoreductase activity, oxidizing metal ions	7.59 × 10^−7^
GO:0042605	peptide antigen binding	1.94 × 10^−6^
GO:0004322	ferroxidase activity	7.06 × 10^−6^
GO:0046977	TAP binding	7.06 × 10^−6^
**Cellular Component (GO)**		
GO-term	Description	FDR *p*-value
GO:0055037	recycling endosome	1.41 × 10^−10^
GO:0042612	MHC class I protein complex	3.39 × 10^−10^
GO:0005769	early endosome	1.94 × 10^−8^
GO:0009986	cell surface	2.09 × 10^−8^
GO:1990712	HFE-transferrin receptor complex	3.87 × 10^−8^
**KEGG Pathways**		
Pathway ID	Description	FDR *p*-value
hsa04978	mineral absorption	1.25 × 10^−15^
hsa04216	ferroptosis	1.75 × 10^−14^
hsa04612	antigen processing and presentation	3.69 × 10^−7^
hsa05330	allograft rejection	1.60 × 10^−6^
hsa05332	graft-versus-host disease	1.60 × 10^−6^
**PFAM Protein Domains**		
Domain	Description	FDR *p*-value
PF07654	immunoglobulin C1-set domain	1.98 × 10^−9^
PF06623	MHC_I C-terminus	2.78 × 10^−9^
PF00129	Class I histocompatibility antigen, domains alpha 1 and 2	2.78 × 10^−9^
PF00210	ferritin-like domain	0.00025
PF07731	multicopper oxidase	0.00025

Shown are *p*-values corrected for multiple testing within each category using the Benjamini–Hochberg procedure (this measure describes how significant the enrichment is). FDR, false discovery rate; GO, gene ontology; HFE, homeostatic iron regulator; KEGG, Kyoto Encyclopedia of Genes and Genome; MHC, major histocompatibility complex; PFAM, Protein Families database; TAP, antigen peptide transporter 1.

**Table 3:** Identification of hub-proteins based on network topology.

Protein Name	Degree Centrality	Betweenness Centrality	Eigenvector Centrality	Subgraph Centrality	Average Score †
HFE	22	44.85	1.00	215,209.80	0.831272727
TFRC	20	30.41	0.99	195,055.82	0.9057
B2M	18	38.04	0.78	97,724.47	0.955555556
SLC11A2	18	16.37	0.88	178,173.90	0.852666667
FTH1	16	34.26	0.64	87,931.41	0.872375
HEPH	16	35.32	0.78	140,287.68	0.857
SLC40A1	16	7.12	0.85	159,359.04	0.882625
CP	12	15.58	0.52	54,947.33	0.906166667
HAMP	12	0.33	0.73	111,523.40	0.889166667
CYBRD1	10	0.00	0.55	78,214.76	0.8214

† All scores rank from 0 to 1, with 1 being the highest possible confidence. A score of 0.5 would indicate that roughly every second interaction might be erroneous (i.e., a false positive). B2M, beta-2 microglobulin; HFE, homeostatic iron regulator; TFRC, transferrin receptor protein 1; HEPH, hephaestin; CP, ceruloplasmin; HAMP, hepcidin.

**Table 4:** Contrasting identified biomarkers to experimentally and manually curated evidence.

Protein	ImmunomeBase IKB	InnateDB Interactions	BiomarkerBase™		MarkerDB	Normal	Abnormal	Exercise
			CTs	Conditions				
HFE	Yes	16	19	988	Yes *	G/G C/C	C282Y (A/G, A/A) H63D (C/G, G/G)	★★★★★
TFRC	Yes	73	146	849	Yes	F †: 1.9–4.4 mg·L^−1^ M †: 2.2–5 mg·L^−1^	F: >4.4 mg·L^−1^ M: >5 mg·L^−1^	★★★★★
B2M	Yes	188	176	946	Yes	1.21–2.7 μg·mL^−1^	>4 μg·mL^−1^	★★
SLC11A2	No	8	3	338	No	258/258 bp and 258 bp alleles overrepresented in athletes		★★★
FTH1	Yes	54	886	1176	Yes	F: 11–307 μg·L^−1^ M: 24–336 μg·L^−1^	F: <11 μg·L^−1^ M: <24 μg·L^−1^	★★★★★
HEPH	No	-	0	115	No	NA	NA	**?**
SLC40A1	No	-	9	513	Yes *	C/C	R178Q (C/T)	★★★
CP	No	7	53	1020	Yes	200–350 mg·L^−1^	<200 mg·L^−1^	**?**
HAMP	Yes	5	173	669	Yes *	C/C F: 1–4.1 nM F ‡: 3.2–8.5 nM M: 1–7.8 nM	C72Ter (C/A, C/T) >8.5 nM	★★★★★
CYBRD1	No	3	0	59	No	NA	NA	**?**

ImmunomeBase contains information about immune-related proteins and is part of the Immunome Knowledge Base (IKB). The IKB does not include proteins specific to the adaptive immune response (e.g., immunoglobulins, T-cell receptors, and major histocompatibility complex). InnateDB is a database that captures an improved coverage of the innate immunity interactome by integrating known interactions and pathways from major public databases together with manually curated data into a centralized resource. BiomarkerBase™ is a commercial resource that exclusively lists every molecular biomarker in active clinical use, and tracks biomarker usage in clinical trials (CTs) across different conditions. MarkerDB is a freely available electronic database that attempts to consolidate information on all known clinical, and a selected set of pre-clinical, biomarkers into a single resource. Literature-based verification was used to report the biomarker use in exercise. F: female; M: male; NA: not available. * Genetic marker; † People of African descent and those residing at 1600 m above sea level were found to have a 6% higher normal value (these differences were additive); ‡ post-menopausal women (55 years of age and older); ★★ weak evidence; ★★★ medium evidence; ★★★★★ strong evidence; **?** not studied.

## Data Availability

The data supporting this review are from previously reported studies and datasets, which have been cited.
